# Anterior Transsternal Approach for Treatment of Upper Thoracic Vertebral Osteomyelitis: Case Report and Review of the Literature

**DOI:** 10.7759/cureus.324

**Published:** 2015-09-16

**Authors:** Hai V Le, Rishi Wadhwa, Praveen Mummaneni, Pierre Theodore

**Affiliations:** 1 Orthopedic Surgery, Massachusetts General Hospital; 2 Orthopaedics, Brigham and Women's Hospital; 3 Department of Neurological Surgery, UCSF Medical Center; 4 Department of Thoracic Surgery, UCSF Medical Center

**Keywords:** transsternal approach, osteomyelitis, cervicothoracic junction, epidural abscess, median sternotomy, spine surgery

## Abstract

Direct ventral access to the cervicothoracic spine (C7-T4) poses a technical challenge in spine surgery, given the vital neurovascular structures residing anterior to the cervicothoracic junction (CTJ). The transsternal approach is a feasible surgical option that allows for direct anterior exposure of the lower cervical and upper thoracic vertebrae. Here, the authors report a case of an elderly gentleman with upper thoracic (T1-2) vertebral osteomyelitis and epidural abscess who underwent a transsternal full median sternotomy for ventral decompression and fusion of C7-T2. We also detail our operative procedure and review relevant literature on different transsternal approaches to the CTJ.

## Introduction

Obtaining direct ventral access to the cervicothoracic spine (C7-T2) for decompression and fusion is technically challenging, given the anatomical constraints. Operative exposure of the cervicothoracic junction (CTJ) is obscured by the skeleton of the thorax (i.e., sternum, clavicles, and ribs). The anterior transsternal approach is a feasible surgical option that allows for direct exposure of the anterior vertebral elements of the CTJ. With a transsternal approach, there are many vital neurovascular structures close by, so great care needs to be carried out while dissecting away the vascular compartment of the superior mediastinum to achieve adequate exposure. Anatomical structures at risk for injury include structures within the carotid sheath, trachea, esophagus, recurrent laryngeal nerves, great vessels, vertebral arteries, and sympathetic trunk [1-6].

Here, the authors report a case of an elderly gentleman with upper thoracic vertebral osteomyelitis (T1-2) and epidural abscess who underwent a transsternal full median sternotomy surgical approach for ventral decompression and fusion of C7-T2. We also detail our operative procedure and review relevant literature on different transsternal approaches to the CTJ. 

## Case presentation

Informed patient consent was obtained for this patient's treatment.

### History

**Figure 1:**
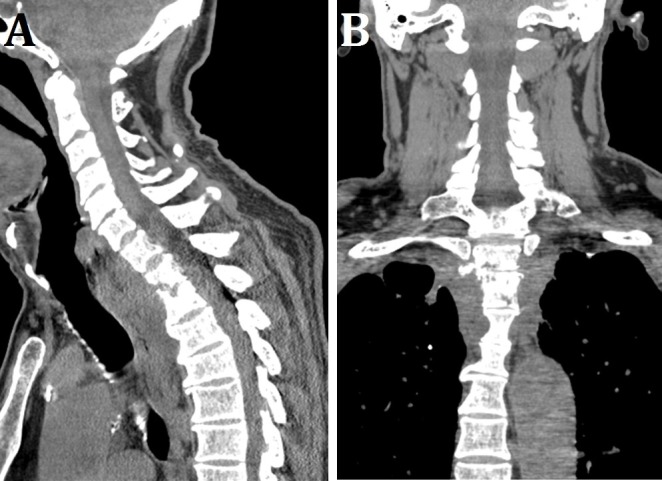
Preoperative CT Imaging ​Preoperative (A) sagittal and (B) coronal CT imaging showing erosions of the T1 and T2 vertebral bodies, with loss of the intervertebral disc space, consistent with discitis and osteomyelitis.

The patient was a 68-year-old diabetic gentleman with a recent history of methicillin-resistant *Staphylococcus aureus* (MRSA) bacteremia secondary to pneumonia, managed with one course of antibiotics. He presented to the emergency department (ED) from an outside hospital for neck pain. In the ED, the patient was afebrile with normal vital signs. Physical exam was within normal limit except for pain on palpation at the neck and right deltoid. Sensory and motor exam were intact. He had an initial WBC count of 13.5, and blood cultures were MRSA positive. Preoperative CT imaging showed erosions of the T1 and T2 vertebral bodies with loss of the intervertebral disc space, consistent with discitis and osteomyelitis. There was evidence of prominent paravertebral soft tissue abnormality, compatible with extension of infection into adjacent soft tissues (Figure 1).

**Figure 2:**
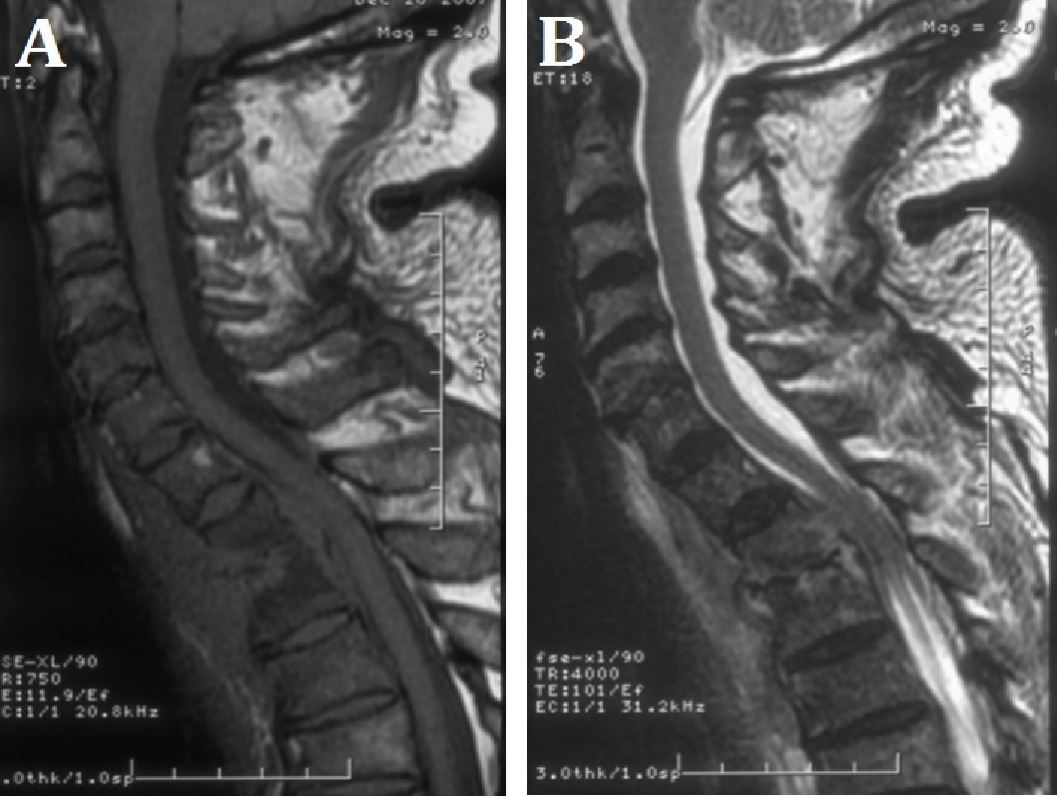
Preoperative MRI Imaging Preoperative sagittal (A) T1- and (B) T2-weighted MRI showing discitis and osteomyelitis at T1 and T2, anterior epidural collection extending from C7-T2, and severe canal stenosis and cord compression.

Preoperative MRI showed discitis and osteomyelitis at T1 and T2, anterior epidural collection extending from C7-T2, and severe canal stenosis and cord compression (Figure 2).

The patient was diagnosed with T1 and T2 osteomyelitis with an epidural abscess causing cord compression. Vancomycin and gentamicin were started, and Neurosurgery Service was consulted for surgical decompression.

### Operation

The patient was taken to the operating room the next day. Under general anesthesia, the patient was appropriately prepped, draped, and positioned supine. Prophylactic antibiotics were administered. Thoracic Surgery (P.T.) performed the initial transsternal exposure of the cervicothoracic spine. A linear incision on the medial border of the right sternocleidomastoid muscle was made and extended down over the manubrium. Median sternotomy was performed with the sternal saw. The sternocleidomastoid was distracted laterally, and the trachea and esophagus were distracted medially for exposure of the anterior cervicothoracic spine. Next, Neurological Surgery (P.M.) performed T1 corpectomy, T2 partial corpectomy, and C7-T2 anterior interbody arthrodesis and plate fixation. Fluoroscopy was utilized to identify the C7-T2 vertebrae. The C7-T1 and T1-T2 disc spaces were incised, and the discs were removed with pituitary rongeurs and curettes. The T1 and T2 vertebral bodies were noted to be partially collapsed, with hollow cavities and covered with phlegmon. The T1 and the upper 50% of the T2 vertebral bodies were removed. An epidural collection of phlegmon was identified and evacuated. Phlegmon adherent to the dura was carefully removed using microdissection techniques. The thecal sac was freed up, and the spinal cord from C7-T2 was decompressed. Foraminotomies were also performed. Next, the surgical site was thoroughly irrigated with fluids containing vancomycin and gentamycin. A mesh cage filled with autograft bone marrow from the sternum was tamped into position to create an anterior arthrodesis and fusion from C7-T2. A cervical plate was drilled and tapped in place using 5 mm variable screws. Hemostasis was achieved with the bipolar, and a Jackson-Pratt drain was placed. Closure of the sternum was performed with No. 6 sternal wires, and the skin closure was performed in the normal fashion. There was no intraoperative complication and estimated blood loss (EBL) was 500 cc.

### Postoperative course

**Figure 3:**
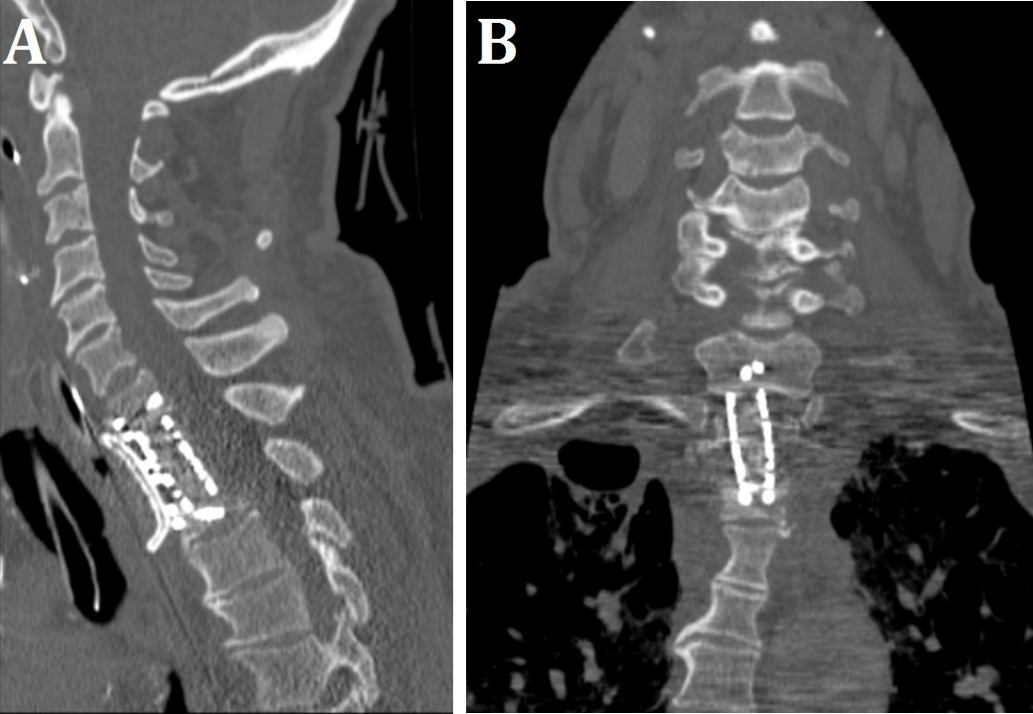
Postoperative CT Imaging Postoperative (A) sagittal and (B) coronal CT imaging showing interval partial corpectomy of T1 and T2 vertebral bodies with anterior spinal fusion extending from C7 through T2 vertebral bodies.

The patient tolerated the above procedure well. Postoperative CT imaging showed interval partial corpectomy of the T1 and T2 vertebral bodies with anterior spinal fusion extending from C7 through T2 vertebral bodies (Figure 3).

Surgical biopsies showed dense plasmacytic infiltrate consistent with osteomyelitis.

## Discussion

As clearly illustrated in this case example, the transsternal full median sternotomy approach offers adequate direct exposure of the lower cervical and upper thoracic vertebrae for surgical treatment of pathology affecting the cervicothoracic spine. Injury to vital neurovascular structures can be avoided with meticulous dissection of the vascular compartment of the superior mediastinum by the thoracic surgeon. Vital structures should be correctly identified, tagged, and gently retracted prior to operating on the spine.

There have been several other clinical studies validating the feasibility of the transsternal approach. Jiang, et al. (2010) evaluated 16 patients who underwent limited sternotomy with a transverse sternal split for the treatment of upper thoracic vertebral tuberculosis (TB). All of the patients tolerated the procedure well and improved postoperatively from a neurological standpoint. There was no incidence of hardware failure or TB recurrence, and the mean time to spinal bone fusion was 4.2 months [7]. Zengming, et al. (2010) studied 54 patients with upper thoracic disease (33 cases of TB, 14 cases of neoplasm, five cases of eosinophilic granuloma, and two cases of traumatic fracture) who underwent anterior decompression and fusion with full median sternotomy. Pain resolved in all of the patients, and motor deficits were improved in patients who initially presented with radiculopathy or myelopathy. There was no serious postoperative approach-related complication [8].

The transsternal approach presented in this article utilizes a full median sternotomy for access of the cervicothoracic spine. However, there have been many modifications to this approach in order to limit extensive osteotomy, such as manubriotomy with clavicle resection, partial lateral manubriotomy, and partial sternotomy with a transverse sternal split [3, 7, 9-13]. There are several potential advantages of a full median sternotomy approach. First of all, it is a technically simpler procedure compared to other modified approaches. Second, it offers better exposure of the mediastinum for improved visualization and manipulation of important neurovascular structures to avoid intraoperative complications. Third, the pectoral girdle is preserved since there is no resection of the clavicle. Lastly, extension of the operative field caudally to as low as T5 can be achieved by dissecting a plane between the brachiocephalic vein, superior vena cava, and ascending aorta [3].

## Conclusions

The transsternal approach utilizing a full median sternotomy can be a safe and effective surgical approach to directly access pathology affecting the lower cervical and upper thoracic vertebrae. Compared to other modified transsternal approaches, the full median sternotomy approach is technically simpler, provides better exposure, preserves the pectoral girdle, and allows for extension of the operative field caudally.
